# Letter to the Editors regarding the article “Patellar resurfacing in primary total knee arthroplasty: a meta-analysis of randomized controlled trials”

**DOI:** 10.1186/s13018-021-02307-7

**Published:** 2021-03-02

**Authors:** Zhong-min Fu, Xiu-mei Tang, Duan Wang, Ning Ning, Zong-ke Zhou

**Affiliations:** 1 West China School of Nursing/Department of orthopedics, West China Hospital, Sichuan University, Chengdu, 610041 People’s Republic of China; 2 Department of Orthopedics, West China Hospital/West China School of Medicine, Sichuan University, Chengdu, 610041 People’s Republic of China

To the Editor:

We read with great interest in the article by Chen et al. [1] regarding the “Patellar resurfacing versus nonresurfacing in total knee arthroplasty: an updated meta-analysis of randomized controlled trials”. We congratulate the authors for publishing their study in this journal. Coincidentally, we did a systematic review on the same topic in December 2020. We appreciate the interesting observation of Chen’s conclusion, and after reading their article, we would like to provide our results for discussion and highlight the difference that remains exploring.

First, the authors searched electronic databases (MEDLINE, Ovid, and Cochrane Library databases), but other unpublished databases like grey literature, which may have more eligible studies were not included. In their study, 32 randomized controlled trials (RCTs) with 6887 knees were analyzed while a total of 52 RCTs (6066 in the patellar resurfacing (PR) group; 7180 in the non-patellar resurfacing (NPR) group) were reported in ours. In their article, Knee Injury and Osteoarthritis Outcome Score (KOOS), Knee Society Score (KSS), function score, noise, revision rate, anterior knee pain (AKP), visual analog score (VAS), range of motion (ROM), Oxford score, Feller score, and patient satisfaction were reported. In our study, surgery length, blood loss, complications (including patellar clunk, patellar crepitus), mortality, and cost-effective analysis were supplied. They did subgroup analysis and divided the patients into two groups: (1) ≤ 3 years and (2) ≥ 5 years. While in our study, subgroup analysis based on short-term results (< 1 year), middle-term (2 to 5 years), and long-term results (6 to 10 years) were reported, and diagnosis-based subgroup analysis was also used.

Second, there existed some difference between the results of Chen and us. the author concluded that the PR group reduced the occurrence of reoperation and noise after surgery, and improved the KSS function scores, while no significant difference in AKP, ROM, Oxford score, KOOS, VAS, Feller score, patellar tilt angle, and the patient satisfaction was found. However, in our study, similar to Teel et al. [2], KSS was not influenced by the two techniques. We also found that the event of AKP was similar in the subgroup of patients with osteoarthritis (OA), while higher in the NPR group in patients with rheumatoid arthritis (RA). Subgroup analysis based on follow-up points found that the PR group had higher KSS scores than the NPR group within 1 year, between 2 and 5 years, while no difference between 6 and 10 years. It suggested the PR group might be more cost-saving than NPR in the short to long term [3–5].

Finally, the author pointed out the limitations of their literature without discussed the selective patellar resurfacing (SPR) which is drawing people’s attention now due to the lack of prospective RCTs. Selective resurfacing may be the main trend in the future which requires more researches on this topic [6]. What is more, a cost-effective analysis was also needed to clarify the benefit of PR in the future [3].

We congratulate the authors for sharing another unique idea again. These results can guide surgeons in making optimal clinical decisions.

## Abbreviations

PR: Patellar resurfacing
NPR: Non-patellar resurfacing
KOOS: Knee Injury and Osteoarthritis Outcome Score
KSS: Knee Society Score
AKP: Anterior knee pain
VAS: Visual analog scale
ROM: Range of motion
OA: Osteoarthritis
RA: Rheumatoid arthritis
